# Anesthetic management for cesarean section in parturient with an uncorrected single ventricle: A case report and literature review

**DOI:** 10.1097/MD.0000000000029421

**Published:** 2022-06-17

**Authors:** Yu Du, Yingzi Yang-Liu, Bin Chen, Ji Wang

**Affiliations:** a Department of Anesthesiology, Nanchong Central Hospital, Second Clinical Medical Institution, North Sichuan Medical College, Nanchong, China; b Department of Anesthesiology, Shifang Maternal and Child Health Hospital, DeYang, China; c Department of Anesthesiology, Affiliated Hospital of North Sichuan Medical College, Nanchong, China.

**Keywords:** anesthesia, cesarean section, pregnancy, single ventricle

## Abstract

**Rationale:**

Patients with a single ventricle, who have not undergone surgery, reportedly have a lower survival rate. Furthermore, multiple pregnancies are rare among these females. We reported a case of anesthesia management of cesarean section in an uncorrected single-ventricular multi-pregnancy woman and review the anesthesia management of the published similar cases.

**Patient concerns:**

An uncorrected single ventricular pregnant woman with a cardiac function of New York Heart Association class II, who had experienced one spontaneous abortion and three vaginal deliveries, was scheduled for cesarean section at 37^+6^ weeks of gestation.

**Diagnoses:**

: Echocardiography revealed a complex congenital heart disease in the mother: a single ventricle (the left ventricle is dominant), atrioventricular valve ectopic, double-inlet left ventricle, abnormal location of the great arteries, probably pulmonary stenosis, atrial septal defect, and left-to-right shunt. The fetus was in breech presentation with umbilical cord around the neck.

**Interventions:**

Cesarean section was successfully performed under the combined spinal epidural anesthesia with careful monitoring.

**Outcomes:**

: Both mother and newborn recovered good and were discharged from the hospital 5 days after surgery without any adverse reactions.

**Lessons:**

Single ventricular pregnant woman with a cardiac function of New York Heart Association class I–II could tolerate pregnancy and delivery well. Both general and regional anesthesia are applicable to cesarean section in these patients. The principle of anesthesia management is to maintain the appropriate balance between systemic vascular resistance and pulmonary vascular resistance, as well as to maintain preload and cardiac output.

## 1 Introduction

Patients with an uncorrected single ventricle rarely survive to adulthood. Pregnancy and delivery rarely occur among these patients. Since single-ventricular pregnancy was first reported in 1963, only a few studies have addressed perioperative anesthesia management in these pregnant women.^[1]^ Systemic and pulmonary circulations originate from a single ventricle, where saturated and desaturated blood are mixed, leading to various degrees of systemic arterial desaturation.^[2]^ Pregnancy and its accompanying hemodynamic alterations increase the burden on this ventricle, resulting in a significantly increased risk of maternal morbidity or mortality.^[3]^

## 2 Case report

The publication of this case was approved by the Ethics Committee of the Affiliated Hospital of North Sichuan Medical College, and written consent was signed by the patient for the purpose of research and publication.

**Figure 1:**
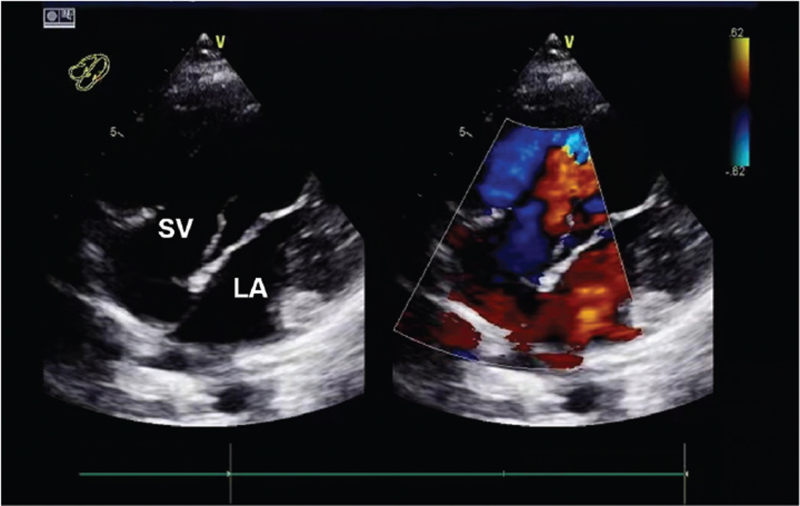
Parasternal long axis view of transthoracic echocardiography showing a single ventricle where the left ventricle is dominant. LA = left atrium, SV = single ventricle.

A 35-year-old woman (height: 152 cm; body weight: 70 kg; New York Heart Association [NYHA] classification: II) was hospitalized at 37^+6^ weeks of pregnancy. She was diagnosed with congenital heart disease (CHD) at the age of 10 years and received no particular treatment. The patient was a gravida 5, para 0, abortion 1 female. She had given birth to three live children through vaginal delivery and experienced one spontaneous abortion. The physical examination revealed a heart rate (HR) of 90 beats/min, blood pressure (BP) of 130/84 mm Hg, respiratory rate of 21 breaths/min, oxygen saturation of 85%, and body temperature of 36.6°C. The arterial blood gas analysis (ABG) before oxygen therapy showed a pH of 7.46, partial pressure of carbon dioxide (PaCO_2_) of 28.0 mm Hg, and partial pressure of oxygen (PaO_2_) of 49.0 mm Hg. Her biochemical examination results, including routine blood tests, coagulation, hepatic and renal functions, serum electrolytes, myocardial damage markers, and brain natriuretic peptide, were normal. The patient reported no particular discomfort during the first and second trimesters, but she recently complained of reduced activity tolerance. As shown in Figure 1, echocardiography revealed a complex CHD. Specifically, there were findings of a single ventricle (the left ventricle is dominant), atrioventricular valve ectopic, double-inlet left ventricle, abnormal location of the great arteries, probably pulmonary stenosis (PS), atrial septal defect, and left-to-right shunt. A fetal ultrasound revealed a live intrauterine fetus with a breech presentation, who was small for gestational age and was wrapped by an umbilical cord around the neck for one circle. Based on the consensus of the multidisciplinary conference, the patient was scheduled for a cesarean section under regional anesthesia.

After entering the operating room, the patient received 100% oxygen via a facemask. Her vital signs before anesthesia included a HR of 105 beats/min, BP of 154/87 mm Hg, and oxygen saturation of 91%. Left radial arterial cannulation was then performed under local anesthesia to monitor BP dynamically. Prior to anesthesia, the ABG revealed a pH of 7.46, PaCO_2_ of 26 mm Hg, PaO_2_ of 55 mm Hg, arterial oxygen saturation of 88.7%, hemoglobin of 117 g/L, hematocrit of 38%, base excess in the extracellular fluid compartment of −5.3 mmol/L, bicarbonate (HCO_3_^−^) levels of 18.5 mmol/L, lactate levels of 1.7 mmol/L, potassium (K^+^) levels of 3.6 mmol/L, and calcium (Ca^2+^) levels of 1.11 mmol/L.

After hydration with 300 mL Ringer's lactate, a combined spinal epidural anesthesia was performed in the left lying position with 2.0 mL of 0.67% ropivacaine, administered intrathecally at the L2-3 intervertebral space. This was followed by insertion of a 3-cm multiorifice epidural catheter. The patient was then placed in the supine position with a left tilt of 20°. Surgery began when the bilateral dermatomal-blockade level reached T4. Three minutes later, a 2.55-kg newborn girl was delivered. Once the umbilical cord was clamped, 100 μg of carbetocin was slowly injected to provide an adequate uterine tone. The maternal hemodynamics were generally stable, but she experienced a brief episode of postural hypotension (systolic BP reached as low as 85 mm Hg) upon changing her position from left tilt to supine prior to surgery. However, her hypotensive episode lasted less than 2 minutes because it was quickly corrected with intravenous ephedrine. The newborn maintained an APGAR score of 10 from 1 to 10 minutes after birth. The instant umbilical cord ABG revealed a pH of 7.37.

The surgical procedure lasted 80 minutes, and the total amounts of fluid input and output were 500 mL and 700 mL (400 mL of urine and 300 mL of estimated blood loss), respectively. At the end of the surgery, the ABG revealed a pH of 7.44, PaCO_2_ of 25 mm Hg, PaO_2_ of 69 mm Hg, arterial oxygen saturation of 95.4%, hemoglobin of 126 g/L, hematocrit of 39%, extracellular fluid compartment of −7.2 mmol/L, HCO_3_^−^ of 17 mmol/L, lactate levels of 2.7 mmol/L, K^+^ of 3.7 mmol/L, and Ca^2+^ of 1.07 mmol/L. Postoperative pain relief was achieved by patient-controlled intravenous analgesia containing sufentanil and butorphanol. She and her baby were discharged from the hospital 5 days later. No congenital cardiac anomalies were detected in the baby.

## 3 Discussion and conclusions

A single ventricle or univentricular heart accounts for approximately 1% of CHD.^[4]^ Successful pregnancy and delivery are rare in uncorrected cases. A literature search was conducted in PubMed (https://www.ncbi.nlm.nih.gov/pubmed/) using the combination strategy with the following keywords: (pregnancy) AND ((single ventricle) OR (univentricular heart)) AND ((anesthesia) OR (cesarean section)). All available cases were summarized, and only 16 pregnant women with an uncorrected single ventricle underwent cesarean section, with 17 pregnancies (Table 1). They almost all are case reports.

**Table 1:** Published case reports: cesarean section in parturient with uncorrected single ventricle.

Author	Anomaly	Age	Delivery time	NYHA	Exercise intolerance	Pulmonary hypertension	Cyanosis	Heart failure	SPO_2_	Anesthesia	Outcome
Yuzpe^[5]^	Single ventricle, laevo-TGA, PS, PDA, hypoplastic aortic arch	17	G1P0, 40W	NA	Yes	No	Yes	No	69%	General: sodium pentothal, nitrous oxide	M-good N-live, 2150 g
Leibbrandt^[6]^	Single ventricle, TGA, small PDA, mild aortic incompetence	2529	^∗^G2P0A1, 40W^∗^G4P1A2, 37W	NAII–III	ProbablyYes	NoYes	NoYes	NoNo	∼84.5%84%	Epidural: NAEpidural: NA	M-good N-live,1540 gM-good N-live, 1520 g
Stiller^[7]^	Single ventricle, subvalvular PS, TGA	23	G1P0, 36W	NA	Yes	No	Yes	No	85%	General anesthesia transferred from epidural anesthesia: NA	M-good N-live, 2353 g
Baumann^[8]^	Single ventricle, TGA, VSD, mitral stenosis	NA	NA, 38W	II–III	Yes	Yes	Yes	No	86%	Epidural: NA	M-good N-live, 2240 g
Tibaldi^[9]^	Single ventricle, TGA, VSD, probably even Eisenmenger syndrome	31	NA, 34W	NA	Probably	Yes	Probably	No	NA	General: NA	M-good N-live, 820 g
Fong^[10]^	Single ventricle, laevo-TGA, ASD, one AV valve, subvalvular PS	29	G4P1A2, 36W	IIa	NA	No	Yes	No	86%	Epidural analgesia: bupivacaineEpidural anesthesia: 3% alkalized 2-chloroprocaine	M-good N-live, 1845 g
Peng^[11]^	Single ventricle, moderate aortic stenosis, PDA	24	NA, 31W	NA	Yes	No	Yes	No	NA	Epidural: NA	M-good N-live, 934 g
Theodoridis^[12]^	Single ventricle, TGA	29	G1P0, 38W	NA	Yes	No	Yes	No	81-97%	Epidural: NA	M-good N-live, 3070 g
Schummer^[13]^	Single ventricle, dextro-TGA, VSD	24	G2P0, 32W	II	Probably	Yes	Yes	No	85%	Epidural: bupivacaine & sufentanil	M-good N-live, 2035 g
Gomez^[14]^	Single ventricle, TGA, PS, VSD	26	G1P0, 27W	II–III	Yes	No	Yes	No	74-78%	Epidural: NA	M-good N-died at 48 h postpartum, 625 g
Boukhris^[15]^	Single ventricle, Eisenmenger syndrome	27	G1P0, 37W	NA	Yes	Yes	Yes	No	70%	Epidural: bupivacaine & sufentanil	M-good N-live, 3000 g
Wei^[16]^	Single ventricle, single atrium, TGA, moderate PS	20	G1P0, 32W	III	Probably	Yes	Yes	No	80-90%	Epidural: NA	M-good N-died 2 days postpartum, 1345 g
Wang^[17]^	Single ventricle, severe pulmonary regurgitation, moderate MR and tricuspid regurgitationSingle ventricle, tricuspid atresia, PS, ASD, PDA, mild MRSingle ventricle, mitral atresia, ASD, severe PS	262034	G3P0A2, 34WG1P0, 34WG2P1, 37W	II–IIIIIIII–III	YesYesYes	NoNoNo	YesYesYes	NoNoNo	80%86% (with oxygen)82%	CSE: NACSE: NAGeneral: NA	M-good N-live, 1330 gM-good N-live, 1460 gM-good N-live, 1600 g
Minicucci^[18]^	Single ventricle, Eisenmenger syndrome	29	G1P0, 31W	NA	Yes	Yes	Yes	No	70%	Spinal anesthesia: bupivacaine & fentanyl; ketamine, i.v.	M-good N-live, 1640 g
Current case	Single ventricle, ASD, ^†^probably PS	35	G5P3A1, 38W	II–III	Yes	No	No	No	85%	CSE: bupivacaine	M-good N-live, 2550 g

ASD = atrial septal defect, CSE = combined spinal epidural, MR = mitral regurgitation, NA = not available, NYHA = New York Heart Association, PDA = patent ductus arteriosus, PS = pulmonary valve stenosis, SPO_2_ = peripheral oxygen saturation TGA = transposition of the great arteries, VSD = ventricular septal defect.

∗ Two pregnancies in one patient.

† Two approximately paralleled great arteries (ID is 22 mm and 18 mm, respectively) emitted from left ventricle; however, the distal end of the arteries are not clear, where the blood flow accelerates in the 18 mm ID artery to its peak flow velocity of 3 m/s and a pressure gradient of 36 mm Hg.

Based on the summary and analysis of the cases, the median maternal age was 26 (range: 17–35) years, and the median gestational age at delivery was 36 (range: 27–40) weeks. Apart from single ventricle, multiple congenital heart anomalies, such as transposition of the great arteries (10/17, 58.82%) and PS (8/17, 47.06%), were commonly detected. Seven patients had pulmonary hypertension (7/17, 41.18%), and three progressed to Eisenmenger syndrome (3/17, 17.65%). Most patients were cyanotic with a median basal oxygen saturation of 84.75% (range: 69%–97%). Except for four patients who received general anesthesia (4/18, 22.22%), all cases were conducted under regional anesthesia (14/18, 77.78%). There were two neonatal deaths (2/18, 11.11%), one died for pulmonary hemorrhage^[14]^ and the other for a premature birth.^[16]^ Although neuraxial block and epidural analgesia reportedly had favorable outcomes for single ventricular women undergoing vaginal delivery, the analgesia management for vaginal delivery is beyond the scope of this article.^[10,19–21]^

The natural history of patients with unoperated single ventricle is poor with a survival rate of ∼30% in the first year of life and a median survival age of 14 years.^[22]^ Several studies have shown that adult patients characterized with double-inlet left ventricular morphology and perfectly balanced circulation with some degree of PS could survive with mild-to-moderate symptomatology and preserved ventricular function.^[22–24]^ In our case, the woman had a similar ventricular morphology and probably PS. She had a preserved cardiac function (NYHA class II) without apparent cyanosis during gestation, and tolerated the multiple labors and delivery.

To achieve good maternal and fetal outcomes, pre-pregnancy counseling as well as peripartum and intrapartum management should be provided by a multidisciplinary management team, including at least one cardiologist, obstetrician, and anesthetist.^[25]^ The anesthetic goal in patients with uncorrected single ventricle should be a balance between systemic vascular resistance and pulmonary vascular resistance (PVR) to avoid intracardiac shunt deterioration. Moreover, the cardiac output (CO) should be stabilized to avoid a forward flow reduction through either pulmonary or systemic circulation. Due to the potential of a right-to-left shunt, peripheral intravenous injection and infusion should minimize the risk of air embolism. Furthermore, appropriate postoperative analgesia contributes a lot to avoiding the catecholamine secretion that increases PVR, reduces pulmonary blood flow, and results in severe cyanosis.^[26]^

Since spinal anesthesia provides a reliable analgesic effect, and the epidural catheter is retained to maintain the anesthesia while extending the surgical time, the combined spinal epidural anesthesia method was chosen. Since regional anesthesia can cause systemic hypotension, fluid infusion was administered in advance, and invasive BP monitoring was performed before anesthesia. The dosage and volume of the intrathecal anesthetics were carefully titrated to maintain the dermatomal level below T4. The patient experienced supine hypotension syndrome after she was placed in the supine position. Then, a vasoconstrictor was immediately administered. The commonly used vasopressors for obstetrical patients include phenylephrine, methoxamine, ephedrine, and norepinephrine. Carbetocin, an oxytocin analog, is routinely administered during cesarean section to reduce postpartum hemorrhage after delivering the fetus. Since oxytocin induces peripheral vasodilatation, hypotension, and increases CO (mediated by an increased HR and stroke volume), oxytocin agents should be infused under the slowest effective dose in single ventricular patients.^[27,28]^ Ergometrine and prostaglandin F analogues (e.g., carboprost) should be avoided in patient with a single ventricle.^[25]^ Besides, fluid balance is an important determinant of a favorable outcome.

In summary, this case showed that pregnant single ventricular women with a NYHA class of I–II could tolerate pregnancy and delivery. Both general and regional anesthesia are applicable to cesarean section in these patients. The principle of anesthesia management is to maintain the appropriate balance between systemic vascular resistance and PVR, and to maintain the preload and CO.

## Author contributions

**Conceptualization:** Ji Wang.

**Data curation:** Yu Du, Yingzi Yang-Liu.

**Methodology:** Yu Du, Bin Chen.

**Writing – original draft:** Yu Du, Yingzi Yang-Liu.

**Writing – review & editing:** Bin Chen, Ji Wang.
